# Evidence for Limited Genetic Compartmentalization of HIV-1 between Lung and Blood

**DOI:** 10.1371/journal.pone.0006949

**Published:** 2009-09-14

**Authors:** Laura Heath, Alan Fox, Jan McClure, Kurt Diem, Angélique B. van 't Wout, Hong Zhao, David R. Park, Jeffrey T. Schouten, Homer L. Twigg, Lawrence Corey, James I. Mullins, John E. Mittler

**Affiliations:** 1 Department of Microbiology, University of Washington, Seattle, Washington, United States of America; 2 Department of Laboratory Medicine, University of Washington, Seattle, Washington, United States of America; 3 Department of Experimental Immunology, Center for Infection and Immunity Amsterdam (CINIMA) at the Academic Medical Center of the University of Amsterdam, Amsterdam, The Netherlands; 4 Pulmonary and Critical Care Medicine, University of Washington, Seattle, Washington, United States of America; 5 General Surgery, University of Washington, Seattle, Washington, United States of America; 6 Division of Pulmonary and Critical Care, Indiana University Medical Center, Indianapolis, Indiana, United States of America; UCLA Medical Center, United States of America

## Abstract

**Background:**

HIV-1 is frequently detected in the lungs of infected individuals and is likely important in the development of pulmonary opportunistic infections. The unique environment of the lung, rich in alveolar macrophages and with specialized local immune responses, may contribute to differential evolution or selection of HIV-1.

**Methodology and Findings:**

We characterized HIV-1 in the lung in relation to contemporaneous viral populations in the blood. The C2-V5 region of HIV-1 *env* was sequenced from paired lung (induced sputum or bronchoalveolar lavage) and blood (plasma RNA and proviral DNA from sorted or unsorted PBMC) from 18 subjects. Compartmentalization between tissue pairs was assessed using 5 established tree or distance-based methods, including permutation tests to determine statistical significance. We found statistical evidence of compartmentalization between lung and blood in 10/18 subjects, although lung and blood sequences were intermingled on phylogenetic trees in all subjects. The subject showing the greatest compartmentalization contained many nearly identical sequences in BAL sample, suggesting clonal expansion may contribute to reduced viral diversity in the lung in some cases. However, HIV-1 sequences in lung were not more homogeneous overall, nor were we able to find a lung-specific genotype associated with macrophage tropism in V3. In all four subjects in whom predicted X4 genotypes were found in blood, predicted X4 genotypes were also found in lung.

**Conclusions:**

Our results support a picture of continuous migration of HIV-1 between circulating blood and lung tissue, with perhaps a very limited degree of localized evolution or clonal replication.

## Introduction

HIV-1 infection is associated with a variety of infectious and non-infectious pulmonary complications, and pulmonary infections appear to accelerate the progression of HIV-related disease [1], [2], [3], [4], [5], [6]. Although pulmonary lymphocytes appear to be the major reservoir for HIV-1 DNA in the lung [7], [8], [9], the lung is distinguished from other organs in having a high percentage of resident macrophages (alveolar macrophage, AM) [10] as potential targets of infection and as sites where HIV replication may occur. Previous studies examining HIV-1 in the lung and blood suggest that lung viruses contain amino acid signatures associated with macrophage tropism [11], have higher percentages of monocytotropic viruses [12], are more likely to be CCR5-tropic [13], and are more homogenous than virus found in blood [11], [14]. These studies all indicate some degree of genetic compartmentalization between lung and blood as well, though the methods used to define compartmentalization varied. In contrast, an autopsy study [15] reported no simple relationship between syncytium-inducing (SI) and non-syncytium-inducing (NSI) genotypes in the lung from three subjects, though they also observed phylogenetic clustering of lung sequences apart from virus in other tissues in two of the three subjects. Genetic compartmentalization has also been observed in C2-C3 *env* sequences from blood and pleural fluid in four of eight HIV-infected subjects with pleural TB [16].

While these studies generally point to genetic compartmentalization between blood and lung, the causes, degree, and extent of compartmentalization have not been fully resolved. The studies cited above all sampled a relatively small number of viruses from a small number of patients, and compartmentalization was not observed in all subjects, nor was it rigorously defined. Certain key studies [11], [14], moreover, focused on a very limited section of the viral genome, the V3 loop. In addition, several of these studies relied on population sequencing, a process that can result in biased estimates of viral diversity and recognition of only major viral genotype(s). Finally, there remains the unresolved issue of why lung viruses should show patterns of compartmentalization consistent with macrophage tropism when most of viral RNA and DNA isolated from the lung comes from pulmonary lymphocytes [8], [9].

In this study, we addressed these issues using a more comprehensive data set: HIV-1 *env* C2-V5 sequences from lung and blood from 18 sets of paired blood and lung samples in which viral sequences were PCR amplified via limiting dilution to avoid viral template resampling and thus obtain an accurate view of variant representation, within the context of the ever-present possibility of biased PCR amplification due to primer selection. The 18 sets consisted of nine pairs of induced sputa (IS) and blood samples and nine pairs of bronchoalveolar lavage (BAL) and blood samples, including four who were sampled before and after antiretroviral therapy. We applied phylogenetic and bioinformatic analyses to these sequence data to test the following hypotheses: (i) Are lung viruses genetically compartmentalized compared to those in the blood? (ii) Does lung preferentially contain viruses of the R5 phenotype? (iii) Does the lung serve as an archival reservoir for viral genotypes present earlier in infection? Contrary to previous reports, which painted a fairly simple picture of viral compartmentalization in the lung, we find a mixed pattern in which virus is compartmentalized in some patients and not compartmentalized in other patients. In all cases where virus did compartmentalize, the effects were subtle; i.e., detectable using statistical tests, though not always immediately obvious from visual inspection of phylogenetic trees. Attempts to relate compartmentalization to diversity, divergence, cell tropism, or clinical indicators such as viral load, CD4+ T cell count, and time infected, did not yield any significant correlations.

## Methods

### Study Populations

Paired lung and blood samples were obtained from three sources: Induced sputum (IS) and bronchoalveolar lavage (BAL) from HIV-infected subjects collected at the University of Washington (UW-BAL), and BAL samples collected from four serially sampled HIV-infected subjects initiating HAART at Indiana University (IU-BAL) with informed consent using human subjects protocols approved by both institutions. Under all three protocols, blood samples were taken within two hours of initiating IS or BAL. All samples came from HIV+ adults (>18 yrs). The IS and UW-BAL samples came from patients with no overt evidence of lung disease. The IU-BAL samples came specifically from subjects with elevated pulmonary lymphocyte counts who did not have evidence of serious pulmonary infections. The IU-BAL patients initiated triple-drug HAART after the initial BAL, and were lavaged again after 4, 24, and 52 weeks. Treatment information and other clinical parameters are summarized in Table 1. The IU-BAL procedures, undertaken in part to address the effects of HAART on pulmonary lymphocytes, have been previously described in detail [17].

**Table 1:** Clinical information of study subjects at time of sampling.

Subject	HIV-RNA	CD4	Time Infected^1^	Therapy at first sample^2^	Smoker	AM Enrichment Procedures	% AM	# IS/BAL sequences	# PL sequences	# PBM sequences	# PBL sequences	# PBMC sequences
IS-8247	<50	517	>8 yr	R, N, S, Z (>5 yr)	N			7	7	12	14	
IS-8835	399,060	7	>8 yr	None	N			12	13	7	13	
IS-8837	64,820	297	>1 yr	None	N			13	14	7	11	
IS-8838	3,610	NA	>8 yr	None	Y			13	11		13	
IS-8840	3,500	1044	>8 yr	None	Y			13	11	10	9	
IS-8886	<50	737	>9 yr	N, Z, L (>4 yr)	Y			10	4	14	14	
IS-8948	56,270	764	>3 yr	None	Y			17	13			18
IS-8992	15,600	NA	>16 yr	None	Y			8	18			11
IS-9000	52	301	∼2.1 yr	E, T, Z, L (4 mo)	NA			8	13			8
IU-BAL1	53,533	361	NA	None	N	Not AM enriched	41	25 BALC				34
IU-BAL2	272,961	313	NA	None	NA	Not AM enriched	10	24 BALC				32
IU-BAL3	31,844	430	NA	None	NA	Not AM enriched	67	24 BALC				33
IU-BAL4	23,794	NA	NA	None	Y	Not AM enriched	92	25 BALC				46
UW-BAL5	141,321	270	∼0.9 yr	None	N	LSM and CD3-depleted	≥84	18 BALC*, 14 BALF	17	7	13	
UW-BAL6	14,642	445	∼5 yr	None^3^	N	LSM and CD3-depleted	≥87	22	16		12	
UW-BAL8	72,691	426	∼10 yr	None^4^	Y	LSM and CD3-depleted	≥91	17 BALC*, 8 BALF	18	6	9	
UW-BAL9	40,304	253	∼20 yr	None	Y	LSM and CD3-depleted	≥99	8	8	10	14	
UW-BAL10	8,001	171	∼1.5 yr	None	N	Percoll gradient prior to CD3 depletion, plus adherence	≥99	14 BALC**, 14 BALC***		11	16	

IS indicates subjects from the induced sputum cohort, IU-BAL indicates subjects from the Indiana University cohort, and UW-BAL indicates subjects from the University of Washington cohort.

1 In cases where exact time was not known, patient was asked to provide an estimate.

2 R  =  ritonavir, N  =  nevirapine, S  =  saquinavir, Z  =  zidovudine, L  =  lamivudine, E  =  efavirenz, T =  tenofovir.

3 Viral load dipped to 400 copies during brief period of HAART 5 months before BAL.

4 Patient had history of HAART reducing VL to undetectable levels in 1997, 2000, and 2001.

### Processing of blood, induced sputum, and BAL

Blood was collected into BD ACD vacutainer tubes (Becton Dickinson 364606) for isolation of plasma (PL) and peripheral blood mononuclear cells (PBMC) by Ficoll density gradient centrifugation using Lymphocyte Separation Medium (LSM) (Cappel 50494, Aurora, OH). At UW, PBMC were fractionated into peripheral blood lymphocytes (PBL) and peripheral blood monocytes (PBM). For the UW IS subjects, monocytes were isolated from PBL using monocyte adherence as described [18]. For the UW BAL subjects, monocytes were isolated from PBL using MACS CD14 microbeads and MACS LS column according to the manufacturer's instructions (Miltenyi Biotec, cat# 130-050-2010).

Induced sputum (IS) were obtained and processed as detailed (Frenkel et al., 2009 submitted). Due to interfering squamous epithelial cells (5–75% of IS cells) in most sputum samples, we fractionated the IS-derived cells using LSM. Alveolar macrophages (AM) and lymphocytes were isolated in the LSM band, and squamous epithelial cells, red blood cells, and dead cells were found in the LSM pellet. We did not attempt isolation of AM from lymphocytes due to low live cell recovery from most induced sputum samples. At all steps in the process small aliquots were taken and cytospins performed for determination of IS cell differential.

Bronchoalveolar lavage (BAL) was performed by advancing a flexible bronchoscope into subsegmental bronchi in the right and left lungs at both UW and IU. Five 30 ml aliquots of sterile 0.9% saline was injected into each subsegment and gently suctioned into a trap [19]. The recovered BAL fluid was passed through a nylon filter to remove clumps and mucus then centrifuged at 300 g for 10 minutes to obtain BAL fluid supernatant (BALF) and BAL cells (BALC). At UW, AM in the BALC were enriched first by fractionation on LSM: AM and lymphocytes were isolated in the band; red blood cells and dead cells were in the pellet. CD3+ lymphocytes were removed from the LSM banded BALC using MACS CD3 microbeads and MACS LC column according to the manufacturer's instructions (Miltenyi Biotec, cat#130-050-101). These are referred to as BALC*. One exception to the above process was BALC from UW-BAL10. In this case BALC were fractionated on 46% Percoll gradients (GE Healthcare, cat# 17089102) prior to CD3+ lymphocyte removal (BALC**). Half of these BALC** were adhered to plastic for 2 hours, rinsed three times with PBS to remove any non-adherent cells, and adherent cells detached with ice-cold PBS-EDTA (BALC***). At all steps in the process small aliquots were taken and cytospins performed for determination of BAL cell differential. BAL fluid supernatant, plasma, and cell pellets were frozen at −80°C for later analysis.

Although the BAL samples were clear on visual inspection, small amounts of blood were detected in the pellets from UW-BAL5 and UW-BAL8. While differential analysis of cytospins showed that the BALC* samples from UW-BAL5 and UW-BAL8 were free of blood lymphocytes, the BALF from these subjects could contain a small amount of viral RNA from plasma.

### PCR and sequencing procedures

Viral RNA was isolated from plasma (PL) and from the BAL fluid of two subjects (BAL05 and BAL08–the two subjects from which we observed a small amount of blood in the BAL pellets) and converted to cDNA as described [20]. Genomic DNA from all other tissues was isolated and purified by the Tissue Protocol of QIAamp®DNA Mini Kit (Qiagen Inc., Valencia, California). DNA and cDNA isolated from patient samples were used to generate PCR products and HIV-1 *env* gp120 sequences of regions C2 through V5 (HXB2 coordinates 6582–7700).

PCR was performed using AmpliTaq® DNA polymerase with GeneAmp® 10X PCR Buffer II and MgCl_2_ solution (Applied Biosystems, Foster City, California), balanced dNTPs at 200 µM (GE Healthcare Life Sciences, Buckinghamshire, U.K.), with outer primers in a first round PCR at 0.3 µM (Invitrogen, Carlsbad, California) -PE1 (5′-TAGAAAGAG CAGAAGACAGTGGCAATGA-3′) and PE2 (5′-GCCTGGAGCTGTTTGATGCCC CA-3′), and inner primers (in a second round PCR) ED5 (5′-ATGGGATCAAAGCCTAAAGCCATGTG-3′) and BH2 (5′-CCTTGGTGGGTGCTACTCCTAATGGTTCA-3′). The PCR conditions used for the first round PCR were an initial 94°C denaturation of 2 minutes, followed by 35 cycles of 94°C for 15 seconds, 55°C for 30 seconds, 72°C for 2 min, concluding with a 72°C extension for 7 min. Conditions used for the second round PCR were identical, except for a cycled extension step of 90 seconds at 72°C. To avoid template re-sampling, we performed limiting dilution PCR, calculating the sample volume necessary to equal one theoretical copy/PCR reaction with the “*Quality*” program (http://ubik.microbiol.washington.edu/computing/quality/jquality.htm) [21],

Independent PCR products were separately cloned using Invitrogen TOPO TA® Cloning kits (Invitrogen Corporation, Carlsbad, California), with each transformation cultured on LB agar, in separate wells of 24-well plates. To prevent template resampling, single colonies were picked from each well and cultured in LB broth overnight, with plasmids isolated using QIAprep spin miniprep kits (QIAGEN Inc.) according to the manufacturer's instructions.

Sequencing was performed by the University of Washington Department of Biochemistry DNA Sequencing Facility (http://depts.washington.edu/biowww/dna/index.html). Before initiating more detailed phylogenetic analyses, we compared sequences against the HIV-1 database using ViroBLAST [22] and grouped them into phylogenetic trees using a neighbor-joining algorithm. Sequences that closely matched viruses in the database or that clustered with sequences from another patient were flagged as possible contaminants.

### Phylogenetic Analysis

Nucleotide sequences were aligned with ClustalW version 1.4 [23] and the alignment refined using MacClade v4.08 software [24]. Version 3.06 of Modeltest [25] was used in conjunction with PAUP* 4.0 [26] to estimate the best evolutionary model for the construction of each maximum likelihood (ML) tree using the Akaike Information Criterion [27]. Each subject alignment was carefully examined for regions of highly variable, ambiguous alignment, in which a preponderance of indels and repeats occurred, making a probable alignment difficult. These regions were excluded on a by-patient basis, while retaining as much of the alignment as possible. PAUP* was used to calculate each patient ML tree with its optimal model from Modeltest in a heuristic search with a neighbor-joining start and SPR branch swapping. Three subtype B reference sequences from the LANL database (accession numbers M63929, U63632, and U95413) were used as outgroup sequences to root the trees. Diversity distances were calculated as the total pair-wise distances in PAUP* between all sequences within each compartment, under the same best-estimated model defined by Modeltest to calculate the tree. Divergence was calculated as the pairwise distance from each sequence to the most recent common ancestor, which is the sequence estimated at the basal root node of the tree node where the outgroup sequences join the ingroup sequences. Statistical comparisons between tissue pairs in each individual were calculated using the Wilcoxon Rank Sums test. Intra- and inter-subject statistical comparisons of diversity between tissue pairs for pooled data were calculated using a statistical that accounts for the inherent dependency of sequences on one another [28] (http://www.scharp.org/users/adecamp/diverstest/runtests.php).

### VESPA analysis and V3 frequency chart

Aligned protein sequences of lung and blood tissue variants were analyzed using Viral Epidemiology Signature Pattern Analysis (VESPA) [29] (http://www.hiv.lanl.gov/content/sequence/VESPA/vespa.html) for each possible pair of tissues. Graphical representations of the pooled lung and pooled blood V3 loops were constructed in WebLogo 3 [30], [31] (http://weblogo.berkeley.edu/).

### Co-receptor Prediction

CCR5 or CXCR4 coreceptor usage was predicted from the amino acid sequence of the V3 loop region using the subtype-B-specific Web PSSM genotypic interpretation algorithm [32] (http://ubik.microbiol.washington.edu/computing/pssm/index.html). Comparative statistics were done using the Fisher's Exact Test.

### Statistical Tests for Compartmentalization

Five methods were used to determine virologic compartmentalization between every tissue pair within each subject [33], [34], [35], [36]. Four of the tests were based on the topology of the phylogenetic trees, while one test relied on genetic distances between sequences. We analyzed each tissue pair separately. The four phylogenetically-derived methods for detecting compartmentalization were: 1) Slatkin-Maddison (SM), which determines the minimum number of migration events between two populations based on the tree topology; 2) Simmonds Association Index (AI), which assesses the degree of population structure, weighting the contribution of each internal node based on how deep it is in the tree, and; Correlation Coefficients, either by 3) length of branches “*r*” or by 4) number of branches “*r^b^*.” The correlation coefficients tests look at any two sequences in a tree to determine whether or not they originate from the same compartment by examining tree structure and distances, i.e., the cumulative genetic distances between sequences (the length of branches) (*r*), or the number of tree branches separating the sequences (*r^b^*). The distance-based method used was the Nearest Neighbor statistic (*Snn*), a measure of how often the “nearest neighbor” (in sequence space) sequences are from the same locality in geographic space. Statistics and compartmentalization tests were implemented in HyPhy as described [37].

Since these methods use different algorithms to measure whether or not there is evidence of genetic compartmentalization between two tissues, they each have various strengths and weaknesses. The SM method is entirely dependent on the structure of the tree, including the number of internal and external branchings as “migration step” counts; however, branch lengths are not taken into account. The AI method also does not take branch lengths into account, and like the SM, is dependent on the structure of the tree, but adds a degree of complexity by weighting the contributions of the nodes between any given two sequences based on where they are located in the tree (i.e. how deep in the tree they are). The correlation coefficients use yet a different equation to assess sequence relatedness; the *r* test actually takes into account branch lengths and the evolutionary paths between sequences, while the *r^b^* examines the number of branches between sequences, and so takes into account population structure in a different manner than the SM and AI tests. While the tree-based methods, as a whole, are dependent on the structure of the tree to varying degrees and are appealing since we are often able to confirm the results when we examine the tree subjectively, the distance-based test is a good indicator of genetic similarity between sequences within a tissue, independent of evolutionary path, and is important to include in the case of a phylogenetic tree being difficult to resolve or having very short branch lengths. While some previous studies have simply examined clustering of specific tissue sequences in phylogenetic trees by eye to classify compartmentalization, sometimes the degree of clustering is not always obvious in trees in which there are multiple small clusters, or in which some tissue sequences cluster together and others are scattered throughout the tree. It is important to have a mathematical method to classify such trees.

We also screened each tissue pair alignment for recombination, which could potentially obscure detection of compartmentalization. We used a genetic algorithm approach [38] implemented in DataMonkey (http://www.datamonkey.org/GARD/) to detect recombination breakpoints. Each non-recombinant fragment defined by these breakpoints was then analyzed separately for compartmentalization using the previous methods.

Associations between compartmentalization and various clinical parameters including diversity, divergence, viral load, and CD4+ T cell count were assessed using Wilcoxon Rank Sums tests, in which subjects exhibiting evidence of compartmentalization between lung and blood were compared to those who did not exhibit evidence of compartmentalization.

We note that none of the statistical tests reported here adjusted for multiple comparisons. While it is customary to adjust *p*-values for multiple comparisons when making positive claims, our use of uncorrected *p*-values is conservative with respect to the negative conclusions in this study (i.e., that compartmentalization was minimal).

### Potential N-Linked glycosylation sites (PNGS) and amino acid length

N-linked glycosylation sites were predicted using N-glycosite [39] (http://www.hiv.lanl.gov/content/hiv-db/GLYCOSITE/glycosite.html). The number of amino acids between HXB2 gp160 position 267 and 471 (C2 to V5) were tallied for each sequence. Statistical comparisons between tissue pairs within each subject were calculated using the Wilcoxon Rank Sums test.

### Nucleotide sequence accession numbers

All sequences were submitted to GenBank and assigned accession numbers GQ444145-GQ444329 and GQ456230-GQ456929.

## Results

### HIV-1 *env* V3 loops from lung and blood samples are indistinguishable

Previous studies [11], [14] claimed that V3 loops from lung samples were more homogeneous in the C-terminal region of the V3 loop but were more diverged overall than blood viruses. To test this finding, we calculated divergence and diversity of full length and C-terminal-region of V3, in lung and blood tissue from all 18 subjects. While there were a few instances in which lung diversity was significantly different from one or more blood tissues within a subject, it was the exception rather than the rule, and overall, lung (from either IS or BAL) was not more or less homogeneous in the whole V3 or in the C-terminal region than any blood tissue. The same held true for the divergence of V3 from the subject's inferred MRCA in the lung and blood (results not shown).

One of these previous studies [11] also claimed that lung BAL contained a highly conserved, negatively charged amino acid motif that may be associated with macrophage tropism [40], while V3 loops in blood were less likely to contain this motif, which was defined as Y-X-T-X-X-X-I-G-D [11] or Y-X-T-X-E-X-I-X-D-I [40] from residues 21 to 29 in the V3 loop. We identified these motifs in both our lung and blood samples in very similar frequencies, regardless of whether the samples were from IS or from confirmed purified BAL (Figure S1). Also, no signature sites in IS or BAL were identified throughout the V3 loop by VESPA, a program designed to detect signature amino acid sequences (see Methods), when compared to any blood tissue (results not shown).

### X4 genotypes are as likely to be found in lung as in blood

**Figure 1:**
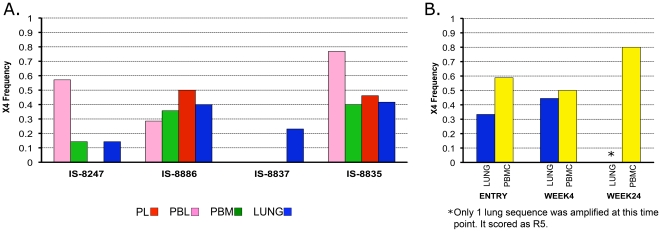
HIV-1 co-receptor usage predictions. X4 viruses were predicted in (A) four IS subjects and (B) on IU subject (IU-BAL4) in multiple timepoints via the WebPSSM. There were no significant differences in frequency of X4 detection between tissues within any subject (Fisher's Exact Test).

Previous studies found that HIV-1 in the lung was more likely to be macrophage- [11] or CCR5-tropic [13] than blood, reflecting different potential target cell pools in each tissue. We applied the WebPSSM algorithm [32] to V3 loops to predict virus coreceptor usage. In all subjects in whom X4 genotypes were detected in blood (3/9 IS subjects and 1/9 BAL samples), X4 viruses were detected in lung samples as well (Figure 1). No single tissue contained a significantly different frequency of X4 viruses than any other tissue. Thus, we saw no evidence that lung-derived virus is enriched for R5 variants relative to blood.

### Statistical evidence for a modest degree of compartmentalization between blood and lung HIV-1 *env* sequences

**Figure 2:**
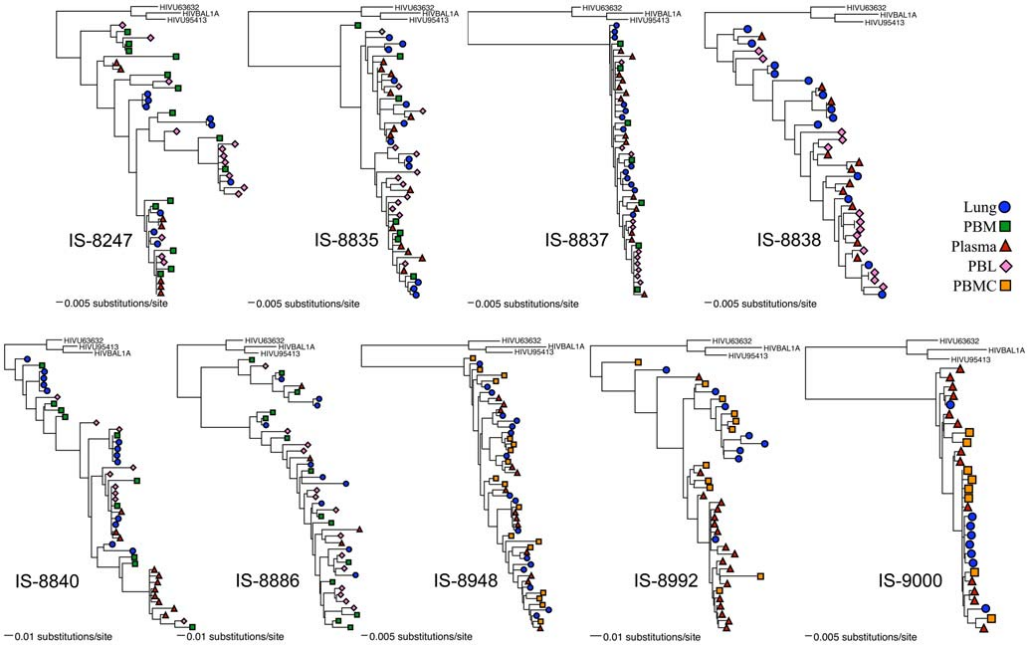
Phylogenetic trees of IS subjects. All trees are maximum likelihood trees calculated under the best-estimated model as determined by ModelTest.

**Figure 3:**
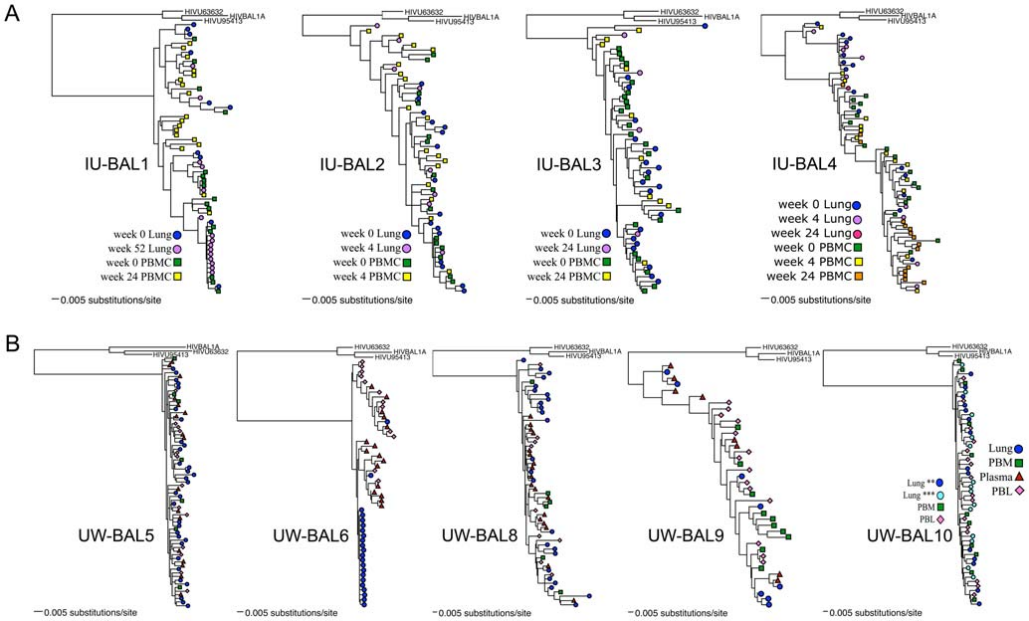
Phylogenetic trees of BAL subjects. All trees are maximum likelihood trees calculated under the best-estimated model as determined by ModelTest. (A) IU-BAL (Indiana University bronchoalveolar lavage) subjects; (B) UW-BAL (University of Washington bronchoalveolar lavage) subjects. ** and ***: alternative methods of AM enrichment in subject UW-BAL10, see methods.

We examined HIV-1 C2-V5 sequences from multiple tissues to assess whether or not there was a restriction of HIV-1 gene flow between the lung and blood. We used five different methods to determine whether or not there was compartmentalization between any two tissues; four of the methods were phylogenetic tree-based (SM, AI, *r*, and *r^b^*) (Figures 2 and 3) while one method (*Snn*) used a genetic distance-based approach, in which distances were derived from nucleotide alignments. Since each method has specific strengths and weaknesses and no gold standard has as yet been accepted, and given the frequency with which these methods frequently disagree [37], for each tissue pair we simply took a majority consensus approach and classified the pair as exhibiting compartmentalization if at least three of the five tests indicated significant evidence for compartmentalization, as determined by the permutation tests employed for each method. We also screened every tissue-pair alignment for recombination with GARD, and re-analyzed the non-recombinatory fragments defined by GARD for compartmentalization in the same manner as before.

Under this design, we found evidence of compartmentalization between lung and at least one blood tissue in six of the nine IS subjects, and in three of the five UW BAL subjects (Table 2). We analyzed each time-point of the IU BAL subjects separately, and did not find evidence of compartmentalization until we examined the non-recombinatory fragments defined by GARD, at which point we found evidence for compartmentalization in the first time-point only of one subject. Recombination breakpoints were found in almost every tissue-pair alignment; however, except in the aforementioned IU BAL case, compartmentalization results were not different from those calculated from the whole alignment (results not shown). We were not able to obtain both IS and BAL samples from the same subject to determine the extent of sampling differences between these two types of tissues, nor were we able to obtain two separate samples within a small window of time (hours or days) from any one subject to examine potential geographic sampling bias effects. We acknowledge that a potentially broader sample was obtained from the BAL, as the IS samples are from the airway and generally had little virus, while the BAL samples encompass an entire lobe of the lung and are likely to come in contact with the epithelial lining fluid as well. However, according to a Fisher's Exact Test, there was not a significant difference in the amount of compartmentalization we saw in the IS subjects (six of nine compartmentalized) vs. the BAL subjects (four of nine compartmentalized).

**Table 2:** Results of compartmentalization tests.

Subject	Tissues from which we obtained sequences	Lung vs. blood tissue pairs in which compartmentalization^1^ was detected	Blood vs. blood tissue pairs in which compartmentalization was detected	SM	*S_nn_*	*r_b_*	*r*	AI
IS-8247	IS, PBL, PBM, PL	Lung vs PL	–	*	***			*
IS-8835	IS, PBL, PBM, PL	–	–	*NS*				
IS-8837	IS, PBL, PBM, PL	Lung vs PBL		**	*	**	**	
		Lung vs PL		*	***		*	
			PBL vs PL	*	*	*	*	
IS-8838	IS, PBL, PL	Lung vs PBL	–		*	*	*	
IS-8840	IS, PBL, PBM, PL	Lung vs PL		***	***	**	**	
			PBL vs PL	*	**	*		
			PBM vs PL	*	*	**	**	
IS-8886	IS, PBL, PBM, PL	–	–	*NS*				
IS-8948	IS, PBMC, PL	–	–	*NS*				
IS-8992	IS, PBMC, PL	Lung vs PL		***	***	**	**	
			PBMC vs PL	**	**	**	**	
IS-9000	IS, PBM, PL	Lung vs PBM	–	*	**		*	
IU-BAL1	BALC^3^, PBMC	–	–	*NS*				
IU-BAL2	BALC^3^, PBMC	–	–	*NS*				
IU-BAL3	BALC^3^, PBMC	–	–	*NS*				
IU-BAL4	BALC^3^, PBMC	Lung vs PBMC	–		*		**	*
UW-BAL5	BALC^4^, BALF^2^, PBL, PBM, PL	–	–	*NS*				
UW-BAL6	BALC^4^, PBL, PL	Lung vs PBL	–	***	***	**	**	*
		Lung vs PL		***	***	**	**	**
UW-BAL8	BALC^4^, BALF, PBL, PBM, PL	Lung (BALC^4^) vs PL		**	**	*	**	
			PBM vs PL		***	*	*	
UW-BAL9	BALC^4^, PBL, PBM, PL	Lung vs PBL		*	**	**	**	
			PBL vs PL	*	**	**	**	
			PBM vs PL		*	**	**	
UW-BAL10	BALC^5^, BALC^6^, PBL, PBM	–	–	*NS*				

Only tissue pairs that were designated as compartmentalized with respect to one another (defined as having significant results in 3/5 tests) are listed.

1 Compartmentalization was designated when significance was detected in at least 3 of the 5 statistical tests.

2 BALF: HIV-RNA from bronchoalveolar lavage fluid.

3 BAL cells not enriched from AM (BALC in Table 1).

4 BAL cells enriched by LSM and CD3-depleted (BALC* in Table 1).

5 BAL cells fractionated on 46% Percoll gradients prior to CD3-depletion (BALC** in Table 1).

6 BAL cells treated as in 5 above, but further enriched for AM by adherence (BALC*** in Table 1).

* *p*-value = 0.01–0.05.

** *p*-value  = 0.001–0.009.

*** *p*-value <0.001 (blank  =  *p*-value >0.05).

*NS* Data not shown, no tissue pair met the criteria for compartmentalization.

Whether or not a subject was determined to have genetic compartmentalization between lung and at least one blood tissue was not related to viral load, CD4 count, or viral diversity or divergence in blood (p>0.05 for all comparisons). Nor was evidence for compartmentalization related to the percent lymphocytes in BAL samples; of the two subjects in which 99% or more of the cells were alveolar macrophages, we found evidence of compartmentalization between lung and blood in one of them but not in the other.

We noted that many of the examples of compartmentalization in Table 2 involved comparisons between DNA (PBM, PBL, PBMC, IS, and BALC) and RNA (PL) sequences. Of the 13 subjects from whom we obtained plasma sequences, we found evidence for compartmentalization between plasma (PL) and lung in 6 subjects. In contrast, we found compartmentalization between PBL and lung in 3 out of 13 subjects for whom we obtained PBL samples; between PBM and lung in 2 out of 10 subjects for whom we obtained PBM samples; and between PBMC and lung in 1 out of 6 subjects for whom we obtained PBMC samples. When we examined compartmentalization outside of the lung (between any two blood tissue pairs, i.e., PBMC, PBL, PBM, PL), we found evidence for compartmentalization between any blood tissue pair in seven comparisons from five subjects, and in all seven cases, PL was one of the blood tissues involved. In no case did we find compartmentalization between just the blood tissues PBL and PBM.

Table 2 also summarizes tests involving RNA from BALF, but these tests are clouded by the fact that there was a trace amount of blood in the two BAL samples from which we detected viral RNA in BALF. Given the plasma viral loads in these subjects, it should be possible to detect viral RNA in BALF even assuming a 100-fold dilution of plasma into BALF. Indeed, the fact that plasma and BALC, but not plasma and BALF, were compartmentalized in UW-BAL8, is consistent with the hypothesis that the RNA in this BALF sample came from plasma.

### Presence of Nearly Identical Sequences in BAL Samples

In two subjects, UW-BAL6 and IU-BAL1 (week 52 timepoint only; there was no matching PBMC, so no compartmentalization tests were done), we noted a large number of nearly identical sequences in BAL, which is something rarely, if ever, seen in blood samples in patients in chronic infection who are not on antiretroviral therapy. We observed this phenomenon in the IU-BAL subject well after the initiation of therapy; we do not know for certain how long this subject had been infected, though it was for at least one year. The UW subject had been on antiretroviral therapy only briefly, was not currently on therapy at the time of sampling, and had been infected for at least five years. Twenty out of 22 lung sequences were identical or nearly identical to one another in this subject, while the blood sequences were not homogeneous (Figure 3B).

### The Diversity And Divergence of HIV-1 *env* Sequences from Lung and Blood Do not Differ Significantly

**Figure 4:**
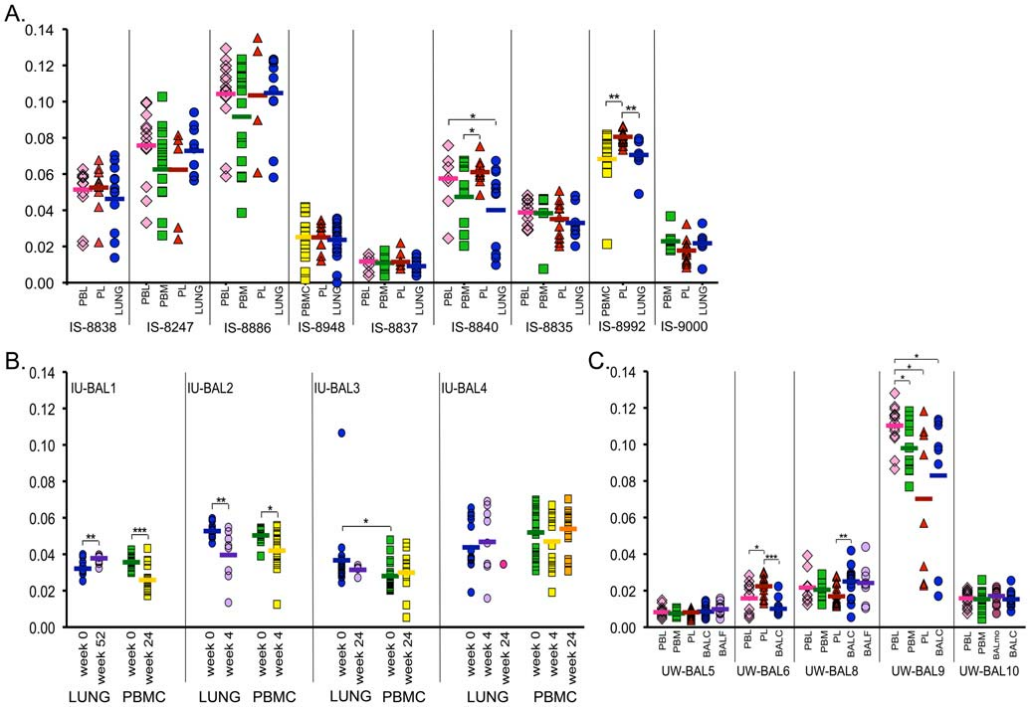
Divergence from MRCA. Distances were calculated under maximum likelihood parameters established under ModelTest. Pair comparisons were made using the Wilcoxon Rank Sums test. (A) IS subjects; (B) IU-BAL subjects; (C) UW-BAL subjects. For pair comparisons: *p-value  = 0.01–0.05; **p-value = 0.001–0.009; ***p-value<0.001.

To assess whether viruses in lung evolve differentially from those in blood, we used the nucleotide alignments to calculate two statistics: (1) divergence, which is the genetic distance between nucleotide sequences from blood and lung to the inferred MRCA (Figure 4); and (2) the mean pairwise nucleotide diversity of viruses in blood and lung samples (Figure S2). In samples from subjects not undergoing anti-retroviral therapy, we observed no consistent trends wherein samples from lung were either more or less divergent or diverse compared to viruses from blood samples. This lack of consistent trend persisted whether lung virus came from IS or BAL cells, whether BAL cells contained >99% AM, whether blood virus came from PBL, PBM, or plasma, or whether the patient had started antiretroviral therapy. Virus from lung was seldom more or less divergent than blood within subjects; lung was significantly more diverged than one blood tissue in two subjects, and significantly less diverged in four subjects, while there was no difference between lung divergence and any blood tissue divergence when divergence values from all subjects were pooled. Likewise, lung was significantly less diverse than virus from a blood compartment in two subjects, and as mentioned above, in one of these subjects many of the lung sequences were identical or nearly identical. Lung was more diverse than at least one blood tissue in five subjects. Overall, when distances from all subjects were pooled, there was no significant difference in diversity between any two tissues.

One subject, IU-BAL3, had one lung sequence that was much more diverged from the MRCA than any other sequence from that subject and was also very diverse compared to the other lung sequences. The distance from this unusual sequence to the MRCA was >0.1 substitutions/site, while the distances to the MRCA of the other sequences were between 0.005 and 0.048 substitutions/site. The alignment was double-checked and a neighbor-joining tree was made containing >200 subtype B reference sequences plus all of the subject sequences, and still the highly diverged sequence grouped closely with the subject's own sequences, and thus there was no evidence that it derived from superinfection from a second source of infection. The sequence was not deemed to be hypermutated, nor did it contain any stop codons. Thus, despite the large distance between it and other sequences, it was not removed or considered to be a contaminant.

### HIV-1 C2-V5 Sequences from Lung and Blood have Similar Numbers of Potential N-Linked Glycosylation Sites and Are the Same Length

The number of Potential N-Linked Glycosylation Sites (PNGS) and sequence length have been examined in the context of HIV-1 tissue compartmentalization [14], [41], [42], [43], with the rationale that the neutralizing antibody response, which influences PNG patterns and accumulations [44], [45], may differ between tissue microenvironments. A PNG, defined as any amino acid sequon N-X-T or N-X-S (unless X = Proline), was calculated for each sequence from HXB2 position 267 to 471 (part of C2 through end of V5). In only a few cases were there differences in the numbers of PNGS between compartments (Figure S3); in the IS cohort, subject 8835 and 8837 sequences from lung had significantly more PNGS than were found in PBL; in subject 8992, sequences from lung had fewer PNGS than did sequences from PL. In the UW BAL cohort, subject BAL5 sequences from the lung had significantly fewer PNGS than sequences from PL, PBL, and PBM. In the IU-BAL cohort, subject BAL4 sequences from lung had significantly more PNGS than PBMC in both the week 0 timepoint and the week 4 timepoint. When subjects were pooled, there was no significant difference in PNGS between any two tissues. The lengths of sequences from each compartment were also very similar, with very little intra-patient variation and no inter-patient variation between compartments (data not shown).

### No Evidence for Directional Viral Gene Flow between Blood and Lung

Given that HIV is not always detected in lung samples and that patients are generally infected via genital fluids and contaminated blood (i.e., by non-aerosol routes), blood should be colonized before lung. To address whether this presumed order of infection affects viral evolution, we examined the phylogenetic trees (Figures 2 and 3) for evidence that blood sequences rooted closer to the MRCA than lung sequences. Visual inspection of the phylogenetic trees and the calculated divergence values (Figure 4) suggested that PBM/PBMC viruses could have been ancestral to IS viruses in donors 8835 and 8992, which was supported by 100% bootstrap values at the ancestral node when sampling with replacement. However, for the remaining IS and BAL samples, there was no evidence for any particular compartment being ancestral to any other, with the minimum genetic divergence in blood and lung tissues being roughly the same and with the basal genotype being roughly equally spread out between the various blood and lung tissues.

## Discussion

Restrictions on the ability of virus to migrate freely between different cells, tissues, and organs can have a profound effect on viral diversity and divergence, the ability of virus to replicate (or persist in a dormant form) during antiretroviral therapy, and the ability of virus to acquire new cell tropisms. Previous studies [11], [14] have suggested that the lung is a privileged site in which HIV-1 evolves quickly towards a homogeneous phenotype. In this study, we have sought to test these predictions by sequencing virus from a large number of paired samples from blood and lung. While we did find evidence for compartmentalization between lung and at least one blood tissue in ten of 18 subjects, compartmentalization was actually quite limited in this data set. In general, HIV-1 variants from the blood and lung were more often intermixed than segregated; in most of the subjects evidence of compartmentalization was not apparent from visual inspection of the phylogenetic trees. Furthermore, we saw no evidence that lung samples contained signature sites in the V3 region or an excess of CCR5-tropic viruses or archival genotypes. The limited evidence of compartmentalization was observed in HIV-1 patients with (Indiana cohort) or without (Seattle cohort) evidence of elevated lung lymphocyte counts, and was not notably influenced by whether virus came from induced sputum, BAL fluid, BAL cells, or BAL cell samples enriched for AM.

The limited amount of compartmentalization that we did observe seemed to be related to two factors. The first was the unexpected presence of identical or nearly identical sequences in some of the BAL samples. Although identical sequences have been observed in samples from patients on HAART [46], [47], it is rare to find identical *env* sequences from blood samples from patients not on therapy. In the case of UW-BAL-06, 20 of the 22 BAL sequences were identical or nearly identical, despite avoiding resampling and contamination. Many other subjects had from two to six identical or nearly identical lung sequences. The presence of nearly identical sequences contributed substantially to statistical evidence of compartmentalization in these subjects, and suggests that compartmentalization could be due, in part, to small, localized clonal expansions as opposed to restrictions in migration [46], [47].

The second factor underlying compartmentalization in our study was a correlation between compartmentalization and plasma RNA. Detecting differences between any proviral tissue and plasma is not surprising, as virus found in plasma was sequenced from HIV-1 RNA, while the virus found in lung tissue, PBL, PBM, and PBMC were sequenced from viral DNA. It has been shown that virus in plasma may reflect a more contemporary quasispecies, in which the cellular origins of the plasma virus are most likely from an actively replicating cell population which turns over very quickly (perhaps from a small subset of the PBMC pool), while cell-associated DNA may harbor archival species derived from latently infected, long-lived cells within the PBMC pool [48], [49]. These differences in turnover rate could be substantial enough to look like compartmentalization, and could therefore be contributing to the amount of blood/lung compartmentalization detected.

By contrast, we did not find evidence that compartmentalization was related to selection for specific viral variants. If antibody escape mutants were driving compartmentalization between lung and blood we might expect to see differing PNGS and length profiles, as HIV-1 variants in the lung would have access to differing cellular compositions and antibody profiles, and would adapt accordingly. Our finding of both X4 and R5 viruses in lung are consistent with previous studies [15], which found both SI and NSI genotypes in a variety of tissues, including lung (though they also reported tissue-specific viral variants in brain, lung, and testis).

Lung tissue is distinguished from other tissues in having a very high proportion of macrophages, and it has been proposed that selection for infection of macrophages might create distinguishing genetic features of lung virus compared to blood virus [14]. Studies have shown that AM, which are the frontline of defense against pathogens in the lung, can be productively infected by HIV-1 [9], [14], though potentially at low levels [50]. The presence of AM as local targets for HIV-1 infection, along with an environment containing a variety of antimicrobial proteins and peptides that comprise the pulmonary innate immune response [51], could theoretically drive selection for specialized HIV-1 quasispecies within the lung. The fact that HIV-infected AM can survive and produce virus for several weeks *in vitro* further suggests that the lung could contain an excess of archival genotypes (viruses that genetically resemble viruses found in lymphocytes during earlier phases of infection).

However, the above theoretical reasons for expecting that the lung could be a reservoir for distinct viral populations need to be balanced against findings that the frequency of detecting HIV-1 in alveolar lymphocytes is much higher than the frequency of detecting HIV-1 in AM in individuals not undergoing ART [8], [9], which could indicate trafficking of lymphocytes from the peripheral blood to the lung [52]. Furthermore, CD4+ T cells in BAL are infected at similar frequencies to CD4+ T cells found in blood in subjects with chronic infection, and are not massively depleted, as has been found in the GI tract [8]. Also, it is known that both cell-free virions and HIV-infected lymphocytes are capable of trafficking between blood and lung [53], [54], and detection of HIV-1 in AM is more likely in patients with more advanced disease and lower peripheral blood CD4+ T cell counts [9].

HIV-1 variants in lung have previously been found to be similar to virus found in lymphoid tissue in some subjects [13], [36]. The lymphatic and circulatory systems continually circulate cells throughout the body, and could be vehicles for free virus and/or HIV-infected T-cells trafficked between lymph nodes and the lung. These processes may be accelerated as T lymphocytes are trafficked into the lung in response to inflammatory reactions, and indeed, HIV-1 infection induces T-cell alveolitis in the lung during early infection [55], [56]. While the relatively small subset of CD4+ T cells found in the lung are infected in high frequencies, it has been reported that CD8+ T cells, present in high quantities during HIV-induced alveolitis, can also be infected [57]. High densities of CD4+ T-cells or CD8+ T-cells may create microenvironments for rapid spread of viruses between lymphocytes and alveolar macrophages. Whatever the mechanism, our results support a picture of continuous communication between circulating blood and lung tissue, with a limited degree of localized evolution or clonal replication.

We cannot exclude the possibility that the lung could serve as a reservoir for HIV-1 in some cases. For instance, suppression of viral load (such as in subjects on effective therapy) may allow for minority cell populations within the lung to sustain populations of viruses that are distinct from those found in blood. Indeed, it has been shown in pediatric subjects under mostly effective ART (<50 copies/mL with blips <400 copies/mL) that virus in induced sputum evolves more drug resistance mutations than virus obtained from blood, and also that viral *env* gene sequences in induced sputum is more diverged than virus in blood [58]. This suggests that viral suppression may enable detection of low-level replication in the lung in the absence of widespread dissemination of virus from blood, and that effective suppression of virus could allow for restriction of migration and be a complicating factor in treatment. However, we did not see the same pattern in our study; two IS subjects had viral load <50 (IS-8247 and IS-8886) and one IS subject had viral load of 52 copies/mL (IS-9000) due to effective ART (Table 1), and divergence did not differ significantly between lung and blood in any of these subjects (Figure 4). This suggests the increased divergence in lung described here could be a phenomenon associated with early successful treatment and perhaps limited to pediatric subjects, though we acknowledge we did not sample enough subjects on effective ART to investigate this issue thoroughly.

In summary, the extensive intermingling of virus from multiple blood tissues with virus from the lung, the lack of a macrophage-tropic motif or signature site in the V3 region (even in purified lung macrophages), and evidence for X4 viruses in both blood and lung, are consistent with a model in which virus in the blood and lung are frequently exchanged. This is an optimistic result for patient treatment, since the presence of genotypically distinct viruses in different tissues complicates efforts to understand disease progression and optimize initial antiretroviral drug regimens.

## Supporting Information

Figure S1 Inter-subject frequencies of amino acid variants at each site in V3 in lung and blood. Sequences were pooled from all subjects. Plots generated from data within each cohort separately (IS and BAL) or by specific blood tissue (PL, PBM, or PBL) also did not show significant differences between lung and blood. Sites 21, 23, 25, and 27–28 have been associated with macrophage tropism in previous studies [5], [33].
(1.92 MB TIF)

Figure S2 Pairwise diversity. Distances were calculated under maximum likelihood parameters established under ModelTest. Pair comparisons were made using a pooled median diversity test [21]. (A) IS subjects; (B) IU-BAL subjects; (C) UW-BAL subjects. For pair comparisons: *p-value  = 0.01–0.05; **p-value  = 0.001–0.009; ***p-value <0.001.
(4.00 MB TIF)

Figure S3 Potential N-Linked Glycosylation sites. The number of N-X-T and N-X-S (with X not equal to P) sequons was counted in each C2 to V5 amino acid sequence. Pair comparisons were made using the Wilcoxon Rank Sums test. (A) IS subjects; (B) IU-BAL subjects; (C) UW-BAL subjects. For pair comparisons: *p-value  = 0.01–0.05; **p-value  = 0.001–0.009; ***p-value <0.001.
(3.77 MB TIF)
