# Supplementary figures and images for: Evidence for Limited Genetic Compartmentalization of HIV-1 between Lung and Blood
Source: PLoS One. 2009 Sep 14;4(9):e6949. doi: 10.1371/journal.pone.0006949 (PMC2736399; doi:10.1371/journal.pone.0006949)

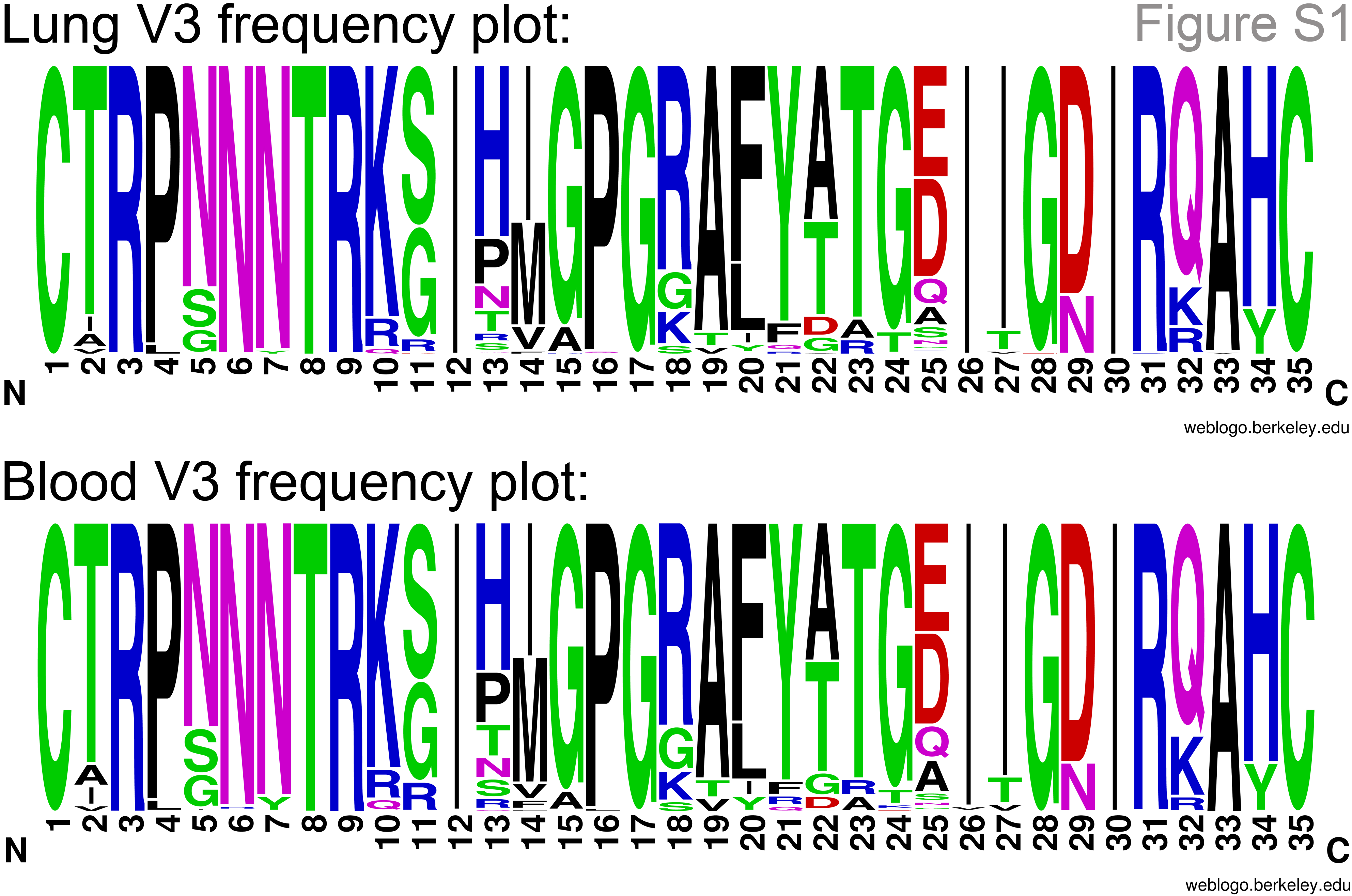

Supplement: Figure S1 — Inter-subject frequencies of amino acid variants at each site in V3 in lung and blood. Sequences were pooled from all subjects. Plots generated from data within each cohort separately (IS and BAL) or by specific blood tissue (PL, PBM, or PBL) also did not show significant differences between lung and blood. Sites 21, 23, 25, and 27–28 have been associated with macrophage tropism in previous studies [5], [33]. (1.92 MB TIF) [file pone.0006949.s001.tif]

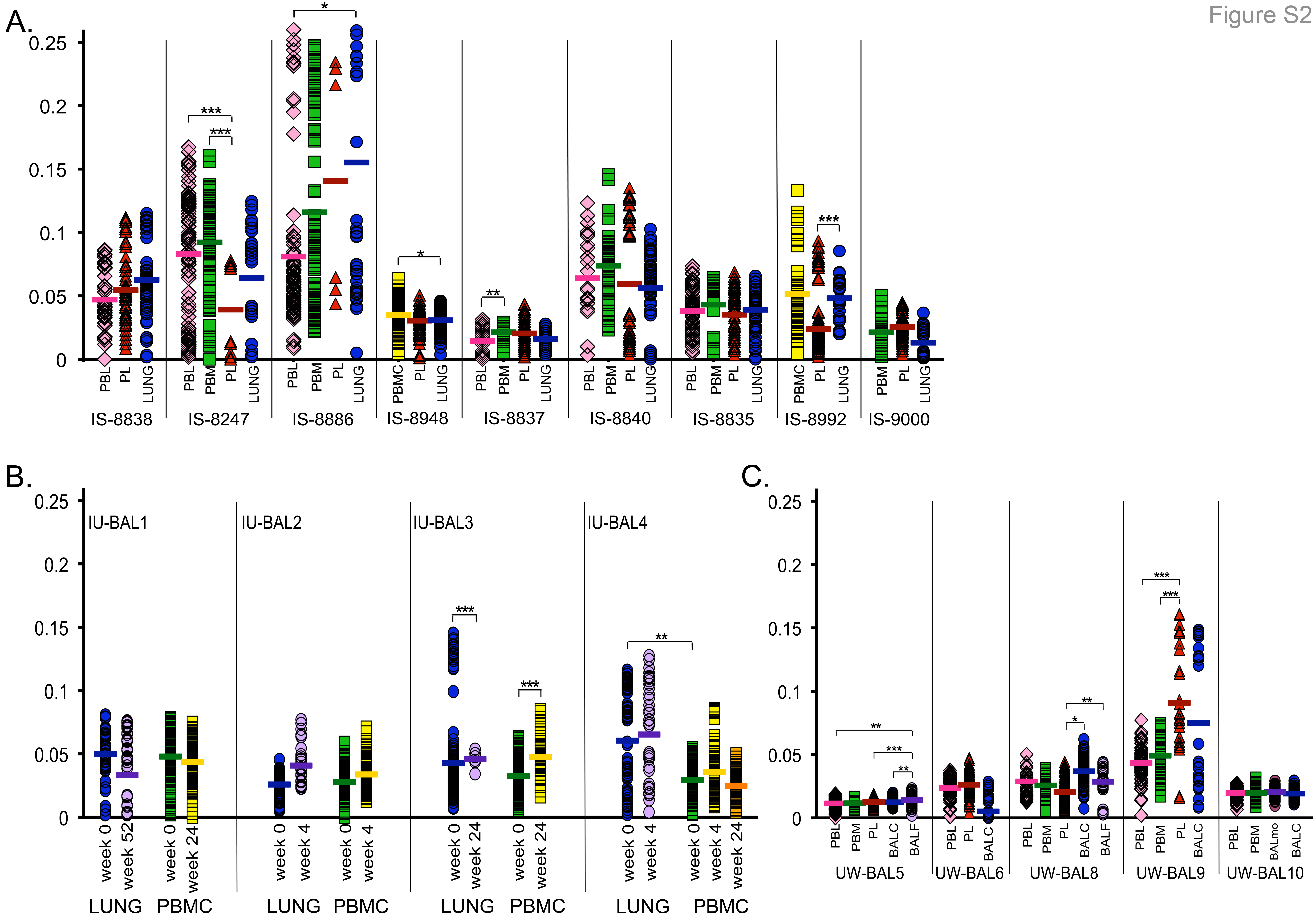

Supplement: Figure S2 — Pairwise diversity. Distances were calculated under maximum likelihood parameters established under ModelTest. Pair comparisons were made using a pooled median diversity test [21]. (A) IS subjects; (B) IU-BAL subjects; (C) UW-BAL subjects. For pair comparisons: *p-value = 0.01–0.05; **p-value = 0.001–0.009; ***p-value <0.001. (4.00 MB TIF) [file pone.0006949.s002.tif]

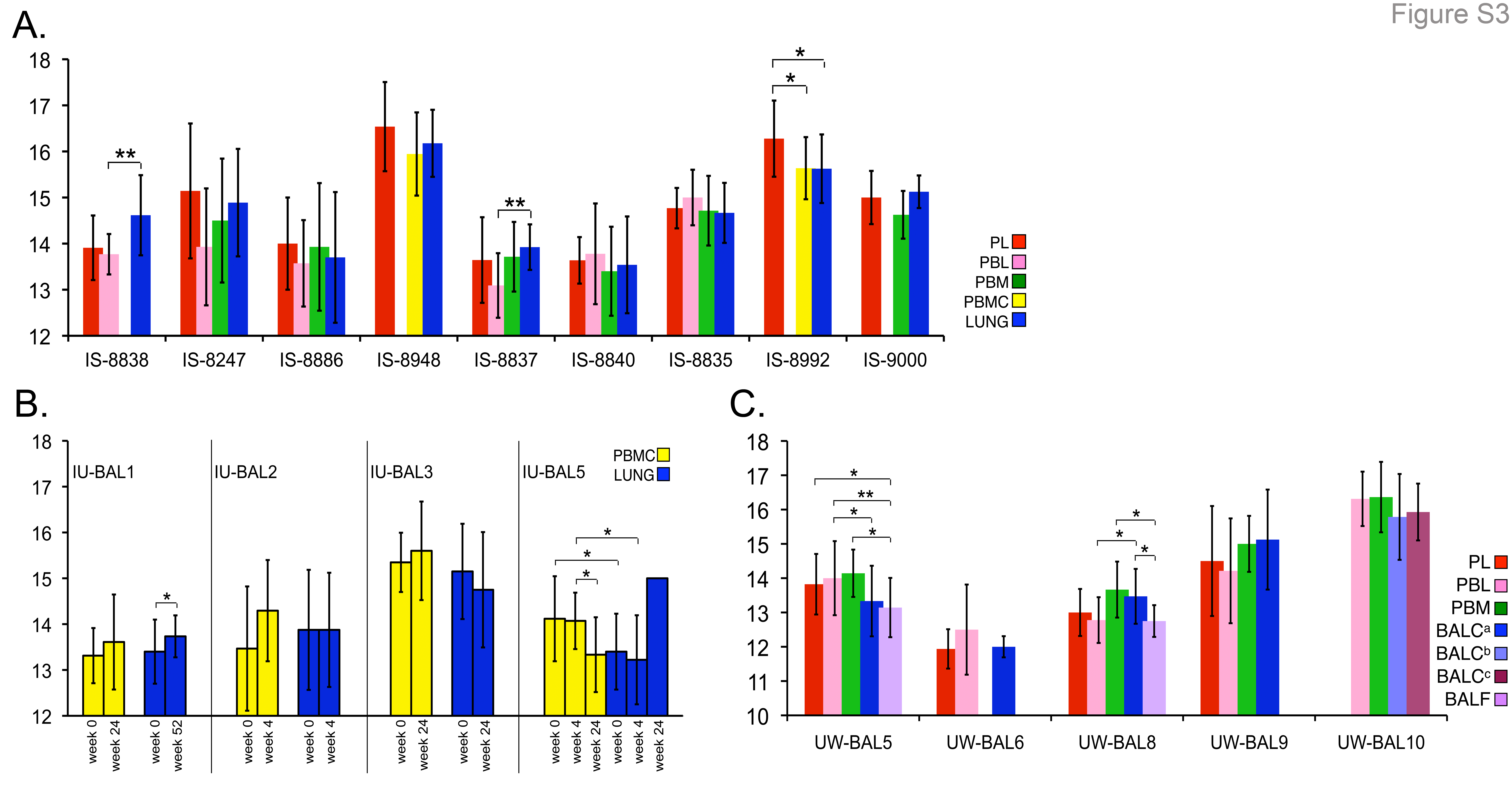

Supplement: Figure S3 — Potential N-Linked Glycosylation sites. The number of N-X-T and N-X-S (with X not equal to P) sequons was counted in each C2 to V5 amino acid sequence. Pair comparisons were made using the Wilcoxon Rank Sums test. (A) IS subjects; (B) IU-BAL subjects; (C) UW-BAL subjects. For pair comparisons: *p-value = 0.01–0.05; **p-value = 0.001–0.009; ***p-value <0.001. (3.77 MB TIF) [file pone.0006949.s003.tif]
